# Psychosocial predictors in upper-extremity vascularized composite allotransplantation: A qualitative study of multidimensional experiences including patients, healthcare professionals, and close relatives

**DOI:** 10.3389/fpsyg.2023.1092725

**Published:** 2023-02-09

**Authors:** Nikolas R. Hummel, Kevin J. Zuo, Simon Talbot, Zoe E. Zimmerman, Jeffrey N. Katz, Sarah E. Kinsley, Martin Kumnig

**Affiliations:** ^1^Department of Psychiatry, Psychotherapy, Psychosomatics and Medical Psychology, Center for Advanced Psychology in Plastic and Transplant Surgery, Medical University of Innsbruck, Innsbruck, Austria; ^2^Department of Orthopaedic Surgery, Division of Hand and Upper Extremity Surgery, Beth Israel Deaconess Medical Center, Boston, MA, United States; ^3^Division of Plastic Surgery, Brigham and Women’s Hospital, Boston, MA, United States; ^4^Department of Orthopaedic Surgery, Brigham and Women’s Hospital, Boston, MA, United States

**Keywords:** vascularized composite allotransplantation, qualitative research, psychosocial predictors, psychosocial outcomes, quality of life

## Abstract

**Background:**

The impact of patient-specific psychosocial factors on functional outcomes after upper-extremity (UE) vascularized composite allotransplantation (VCA) is poorly understood. The objective of this study was to identify relevant psychosocial predictors for success or failure of UE VCA in an Austrian cohort.

**Methods:**

A qualitative study was undertaken consisting of semi-structured interviews with UE VCA staff, transplanted patients, and close relatives. Participants were asked about their perceptions of factors that either favored or hindered a successful transplant outcome, including functional status before surgery, preparation for transplant, decision-making, rehabilitation and functional outcome after surgery, and family and social support. Interviews were conducted online and recorded with the consent of interviewees.

**Results:**

Four bilateral UE VCA patients, 7 healthcare professionals, and a sister of a patient participated in the study. Thematic analysis revealed the importance of an expert interdisciplinary team with adequate resources for patient selection. Psychosocial aspects of prospective candidates are crucial to evaluate as they contribute to success. Both patients and providers may be impacted by public perceptions of UE VCA. Functional outcomes are optimized with a life-long commitment to rehabilitation as well as close, ongoing provider involvement.

**Conclusion:**

Psychosocial factors are important elements in the assessment and follow-up care for UE VCA. To best capture psychosocial elements of care, protocols must be individualized, patient-centered, and interdisciplinary. Investigating psychosocial predictors and collecting outcomes is, thus, critical to justifying UE VCA as a medical intervention and to providing accurate and salient information to prospective candidates.

## Introduction

The primary goal of upper-extremity vascularized composite allotransplantation (UE VCA) is to maximize transplant recipients’ functional, emotional, and social quality of life (QoL). Outcomes are much broader than graft survival, range of motion, and other traditional objective endpoints. Accordingly, while immunological, biomechanical, and medical factors are important determinants of transplant outcomes, psychosocial factors also play critical roles. Due to the small number of performed UE VCA cases and the heterogeneous screening and follow-up protocols, few studies of psychosocial predictors of outcomes have been performed worldwide; hence, our understanding of this topic is limited (Kumnig et al., 2012, 2013; Singh et al., 2015, 2016; Jowsey-Gregoire et al., 2016; Heineman et al., 2020; Bound Alberti et al., 2022; Kumnig et al., 2022), although, several transplant centers worldwide have developed specific VCA programs (Kumnig et al., 2013). Recent research (e.g., Heineman et al., 2020; Kinsley et al., 2022) provides a good understanding of functional and sensory, and psychosocial outcomes (Kumnig et al., 2014). Additionally, comprehensive qualitative research initiatives have recently been undertaken to enhance psychosocial outcomes in VCA and discuss the key psychosocial challenges faced in UE VCA today.

As noted above, UE VCA is life enhancing rather than life saving such as in the case in solid organ transplantation (SOT; Dickenson, 1999; Dubernard, 2011). Hence, the risk-to-benefit ratio is quite different than with SOT in which the risks are offset by the lifesaving nature of the procedure (Tobin et al., 2005; Kumnig et al., 2013, 2014). Ideally, candidates will be strongly motivated for transplantation; have demonstrated reliable adherence with medical care in the past; have strong family support networks; and utilize acceptance, flexibility, and problem solving in adapting to the loss of function from the injury/deficit and for future rehabilitation following transplantation (Sears Jr. et al., 1995; Olbrisch, 1996; Leo et al., 2003; Kumnig et al., 2014). A candidate who has been educated about the various risks, benefits, and demands of the transplant experience, and who has been prepared for the psychological stresses of the experience is more likely to have appropriate expectations regarding the risks of immunosuppression and surgical complications, as well as a more realistic understanding of potential functional gains after transplantation (Lanzetta et al., 2001; Sicard, 2011).

In reality, UE VCA candidates may overestimate the benefits of the procedure while underestimating the surgical risks, duration of recovery, demanding post-transplant medication regimen, and intense rehabilitation requirements (Simmons, 2000; Brenner et al., 2002; Baylis, 2004; Brouha et al., 2006; Petruzzo et al., 2010; Sicard, 2011; Kalluri and Hardinger, 2012; Kumnig et al., 2014; Kinsley et al., 2020). Unmet expectations, an inability to incorporate the transplanted hand (s) (Lanzetta et al., 2005, 2007; Petruzzo et al., 2008; Kumnig et al., 2013), and either new or recurring psychiatric conditions have been reported after UE VCA (Schuind et al., 2007), including suicide attempts (Schuind et al., 2006) and request for amputation (Petruzzo et al., 2008; Petruzzo and Dubernard, 2011). Additionally, recipients may be frustrated with the lengthy process of recovery. The loss of ability to perform tasks that were possible preoperatively also leads to initial postoperative decrease in quality of life (Petruzzo et al., 2010; Kumnig et al., 2014).

Fortunately, the majority of recipients have reported successful psychological integration of the hand(s), and improved confidence in appearance and in social situations (Schuind et al., 2006; Jablecki, 2011). Recipients that have assimilated the transplanted hand(s) into their body-/self-image are generally able to develop a sense of “ownership.” Additional important outcomes are the observed improvements in QOL and activities of daily living (Kumnig et al., 2014). It has become apparent that patients’ coping styles, support from family and friends, and financial factors are important predictors of successful outcomes (Shores, 2011). Recent findings also show that patients’ relationships to healthcare providers, as well as to family members and peers, are correlated with satisfaction (Kinsley et al., 2022). Patients may also experience stress due to media attention which has occurred in a number of UE VCA cases (Kumnig et al., 2012); this impacts the decision to undergo a UE VCA procedure and the post-transplant course. Therefore, multidimensional psychosocial evaluation and follow-up protocols should include these additional domains: health literacy regarding transplantation, assessment of pain related to amputation and phantom limb pain, family support, adaptation to prosthesis, financial and family stressors, assessed through multiple interactions with a variety of assessors including psychiatrists, psychologists, social workers, hand therapists, and all team members (Dobbels et al., 2009; Shores, 2011; Kumnig et al., 2014).

This qualitative study has a similar design as the recently published qualitative investigation of Kinsley et al. (2022), which aimed to explore the role of patient-specific factors through a qualitative analysis of interviews with UE VCA recipients.

In the present study, we have adapted and expanded the interview protocol combining the interviews with those of healthcare professionals of the interdisciplinary VCA team and relatives of the UE VCA recipients and contrasting the outcomes of United States VCA recipients with those in Austria. The main goal of this qualitative research was to elucidate relevant psychosocial predictors for success or failure of UE VCA in this European cohort. By understanding these psychosocial factors, we hope to enhance existing heterogeneous screening and follow-up protocols by including identified important psychosocial factors in the evaluation and peri-operative management and optimization of potential candidates for UE VCA.

## Materials and methods

### Participants

In total five patients underwent reconstructive UE VCA at the Medical University of Innsbruck so far. One of these patients who received unilateral UE VCA in 2009 died due to progressive gastric cancer, leaving a total of four potential patients, which were eligible to be invited to participate in an online interview. Additionally, online interviews with the staff of the Innsbruck VCA team were scheduled to assess the healthcare professionals’ overall expertise working in the field of UE VCA. Also, interviews with close relatives of the four transplanted patients have been planned to gather individual experiences of partners/main social daily contacts living with somebody who underwent UE VCA.

Inclusion criteria consisted of all patients and healthcare team members with direct experience undergoing VCA or providing care for VCA patients. These include all patients who have undergone UE VCA at Innsbruck, close family members of patients such as a spouse or primary caregiver, and all core members of the interdisciplinary transplant team, which includes surgeons, bioethicists, rehabilitation specialists, psychologists, dermatologists, and institutional or administrative leaders. There were no exclusion criteria other than individuals unable or unwilling to provide commentary or participate in the study. All potential participants and staff received an introductory email inviting participation in an online interview. Written informed consent was provided by the final participants.

A total of four bilateral transplanted patients as well as seven healthcare professionals of the Innsbruck VCA team were interviewed. Overall, four close relatives/partners were potentially eligible to take part in an interview; however, only one family member was enrolled in the study. The wife of the first transplanted patient did not provide informed consent (due to missing skills to realize the online interview), the second patient was living alone (without a partner), and the fifth patient lived with his mother who was almost 90 years old. Only the sister of the third patient provided informed consent to participate in this qualitative research study.

All study activities were approved by the Ethics Committee of the Medical University Hospital, Innsbruck (vote 1044/2020). Recruitment and interviews followed a similar process to that at Brigham and Women’s Hospital (Harvard Medical School) published by Kinsley et al. (2022), representing the largest UE VCA samples investigated in this way.

### Interviews

We conducted a total of 12 interviews: 7 with UE VCA healthcare professionals, 4 with UE VCA transplanted patients, and one with a patient’s relative. A trained interviewer conducted the interviews using a semi-structured guide. The interview guide consisted of open-ended questions that asked participants about their perceptions of factors that either favored or hindered a successful transplant outcome. Topics included functional status before surgery, experience with the preparation for transplant, decision process and information transfer, rehabilitation and functional outcome after surgery, and family and social support. Interviews were conducted online and recorded with the consent of interviewees. Subsequently, a qualitative analysis of the interview transcripts was performed.

### Thematic analysis

Thematic analysis followed routinely-accepted methodology in qualitative research (Kinsley et al., 2020). Six researchers collaboratively created a coding scheme consisting of 51 codes and 10 subcodes. The codes consisted of single words or short phrases to produce sections of text that meaningfully related to the study’s guiding question, “What factors influence the success of upper extremity VCA?” These codes were applied to all transcript data by six members of the Innsbruck and Harvard research group.

**Figure 1:**
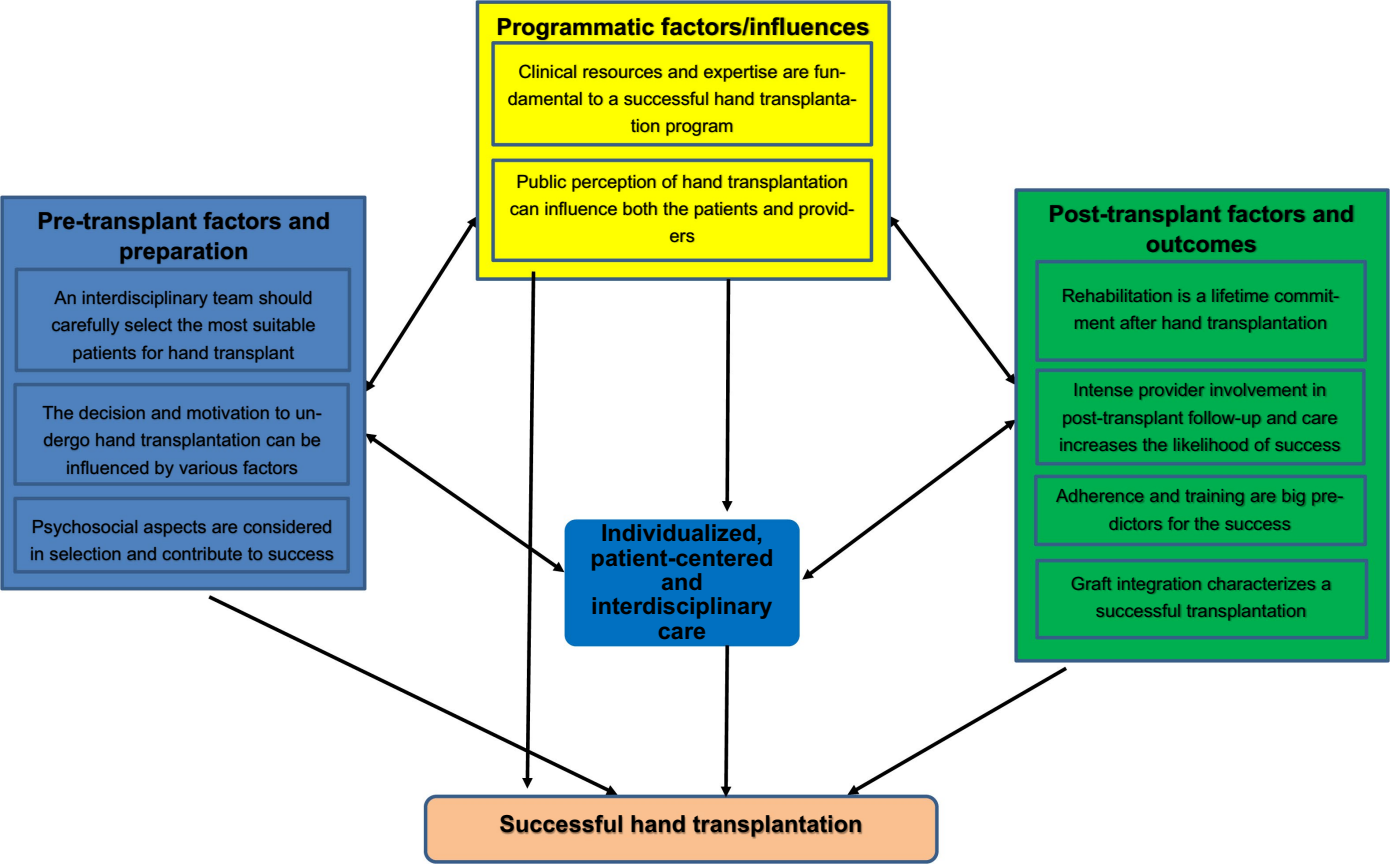
Thematic map.

Members of the Innsbruck team grouped the previously created codes into themes, which were formulated as directed hypotheses. Care was taken to ensure that they had sufficient internal homogeneity and external heterogeneity to be sufficiently grouped or distinct from the other themes. We used qualitative analysis software (Atlas.ti) to extract citations supporting the themes. The Innsbruck team listened to the taped recordings and identified themes, using the qualitative analysis software to index their digital location in the recordings, creating codes that have been connected and grouped to themes. All investigators agreed upon a thematic map showing relationships between the individual themes and the guiding questions of the study (for details please see Figure 1).

## Results

### Participants

The study sample consisted of 4 bilateral upper limb VCA patients, the sister of an upper limb VCA patient, and 7 upper limb VCA healthcare professionals. This latter group consisted of 5 transplant surgeons and 1 rehabilitation physician. Four participants were female and 8 were male.

Thematic analysis of participant commentaries led to the identification of three main psychosocial domains: pre-transplant factors and preparation, programmatic factors and influences, and post-transplant factors and outcomes. For each of these three main domains, individual themes were established; these are summarized in detail below and illustrated in Figure 1. Representative participant quotations for each of the domains and themes are illustrated in Table 1.

**Table 1:** Categories and themes from the thematic map, associated hypotheses and supporting quotations from the interview transcripts.

Categories	Themes	Sample of supporting text from transcripts
Pre-transplant factors and preparation		
**Patient selection**	An interdisciplinary team should carefully select the most suitable patients for hand transplant	*“Mobility is one thing, but I think the main indication for hand transplantation is the sensation that the patients then have in their hands, so that they can feel things and people. And that is what the hand transplant provides, mobility varies greatly depending on compliance and motivation, but a prosthesis can do that too.” (1C)* *“I think transplanting a hand for someone who’s been waiting for it and who says life only gets good if they have their hands transplanted is something to think twice about. (…) After that, he is not disciplined enough to train or perform in such a way that it actually has a benefit for him in terms of movement. (…) But if it’s a patient who says he wants the hands because he wants to feel and he’s not restricted in his life in any way because he has designed his daily routine such that he could theoretically get by without hands, but he really wants to feel, I would say ok. He′s a reflective person who knows exactly why he wants that and who has organized his life in such a way that it also works without hands.” (2C)* *“A congenital malformation is something that should not be transplanted. (..) Then there are patients who have psychological problems, who do not fit into the rehabilitation scheme, who are not compliant, do not perceive control and take medication irregularly, and do not go to occupational therapy. These are things that should definitely be filtered out preoperatively.” (3C)* *It is very important that each discipline sees the patient and then discusses collectively because patients tell different doctors different things. Because, for example, they have a better personal basis for conversation, for example with another doctor. Or you can talk to one doctor very easily about the medication, but you do not dare say that you have a functional problem. Some patients want to impress certain colleagues, while others do not like them. And that’s why interdisciplinarity is so important, because we are all human. (4C)* *“I have the image in my head that we have managed to set up a football team in which everyone is aware that in order to win this match we are a team and we have to stick together.” (5C)*
**Motives**	The decision and motivation to undergo hand transplantation can be influenced by various factors	*“The most important thing is that you have a hand at all and do not walk around with stumps. That was a disaster, that was a real eye-catcher.” (1R)* *“The patient wanted bodily integrity - he was not considered complete in his village community since he has a deficit.” (6C)* *“Even if you hug someone with your hands, it’s flesh and skin as it should be and not plastic.” (2R)* *“Sitting at the inn table and being able to put both hands on the table was certainly more important to him, because he had a functioning hand with sensibility and motor skills, and because of that the optics and aesthetics were most important for the patient.” (7C)* *“It was also an important concern for me, when I stroke my wife’s hair with my hands, whether I will feel it too.” (3R)* *“Sensibility is the main argument for hand transplantation from a professional point of view. Functionally, prostheses can do a lot, only biofeedback is missing, sensibility feedback is missing” (8C)* *“It goes without saying that I had the unspeakable desire to ride a motorcycle again.” (4R)* *“And the second reason was his wife’s Christmas tree plantation, on which he wanted to continue working. And for that he needed a strong, functioning hand, since he was never able to work with his prosthesis.” (9C)*
**Psychological factors**	Psychosocial aspects are considered in selection and contribute to success	*“I would not like to transplant someone who does not have a regular daily routine, someone who has no idea what they would like or could do in the future. (..) So I would like to transplant someone who says I have a job that I go to every day. I have a group of friends that I meet up with regularly. So someone who has very regular routines, who does not live just for the moment, who still lives his life even though he has no hands, is suitable.” (10C)* *“Any kind of addictive behavior in terms of substance use and alcohol should be an absolute contraindication. Smoking should also be an absolute contraindication. (..). Of course, this is a patient who is vulnerable, who perhaps has less self-discipline, who does not have such an orderly life, and I think that should be a contraindication.” (11C)* *When we talk about hand transplants, we must not only assume absolute contraindications. Of course there are. Take someone who has severe dementia and has had a serious accident as a result and lost both hands. (..)*. *Where you simply have to say that this cannot work due to dementia and the lack of cognitive abilities. This is an absolute contraindication.” (12C)* *“I think it’s the most important attitude is the self-discipline. (..) So self-discipline is what brings the maximum benefit to the patient.” (13C)* *“I think family and supporters, who of course were already there before the operation, are crucial. So a catchment area for physical and mental problems in the immediate family and circle of friends is extremely important.” (14C)* *“I think the family should be behind it because the transplant and everything around it does not stop with the transplant, it continues throughout life. (..) I think that it makes the whole situation and the whole project easier when the partner the family, or the parents are behind it and participate, because that promotes compliance.” (15C)*
**Programmatic factors/influences**		
**Clinical resources**	Clinical resources and expertise are fundamental to a successful hand transplantation program	*“The first important thing is specialist knowledge*, i.e.*, I have to know the literature, what are the others doing, what hardware do I need. Between the lines, I need to go where there is a lot of transplanting and talk to other people.” (16C)* *“The technical know-how alone is not enough. I also need resources, I need a structured program, I need team players so that it can work.” (17C)*
**Public perception**	Public perception of hand transplantation can influence both the patients and providers	*“But they always think [hand transplant is] great. The public thinks that’s great. (..) It’s also simply fascinating.” (18C)* *“Before the transplant, your brother received attention in a negative sense, so you have lost something, you are handicapped, you are limited, you look different. And through the hand transplant, you get attention from the outside, but weighted more positively, in the sense that something special has been done.” (19C)* *“We doctors also make a lot of mistakes, (..) [but instead of reflecting them] we try to surpass each other with the most amazing and spectacular operations. (..) This also provokes a certain reaction and fear in society. If this becomes a routine procedure, (..) it will no longer be so sensational and you can no longer satisfy the media with it, but it is more of a reassurance for the patients. That’s why I’m a fan of standardization.” (20C)*
**Post-transplant factors and outcomes**		
**Rehabilitation**	Rehabilitation is a lifetime commitment after hand transplantation	*“You have to mention again and again that this is a long road that can be paved with complications. (21C)* *“The most important thing is good physiotherapy and rehabilitation. (22C)* *He still reports improvement. That has never stagnated. (..) I know that the patient was always motivated to work with his hands.” (23C)* *“Not a year or two years. You have to work a lot. You have to know that it will be a very difficult road, hard work.” (5R)* *“This was a patient, who had a high level of activities of daily living (ADLs), and that is also important. And this is also important for the rehabilitation phase.” (24C)* *“In the beginning, I think it’s normal to make huge moving progress. If you follow the measurements of physical therapy or occupational therapy, there are slight but measurable noticeable improvements every year. In the beginning, the successes were great, big steps, but also changes are apparent year after year, even today. Improvements can be seen in terms of strength, grip, feeling, warmth and perception of cold.” (25C)*
**Follow-up and care**	Intense provider involvement in post-transplant follow-up and care increases the likelihood of success	*“You have to be able to work with the patients, in the sense that they have to enter into a partnership with the doctor who treats them. (..) You need even more trust than usual in doctor-patient relationships. The transplant patients have to report quickly if something does not fit. The doctor must be available. Such a patient is a task that requires a team. That demands a lot from the medical staff. If you are not willing to do this, you will not get good results.” (…) (26C)* *It takes someone willing to deal with these patients 24 h a day, 7 days a week, 365 days a year. (..) If these patients have a problem, it can very quickly end in a downward spiral.” (27C)* *“I find the support to be very, very time-consuming. The patients require an extremely large amount of time and effort (..)You go on vacation and then you get the messages and the phone call while you are on vacation. (..) that is very time-consuming. Because no finding should be overlooked or forgotten.” (28C)*
**Adherence and training**	Adherence and training are big predictors for the success	*“You have to trust the doctors 100% and do everything the doctors say. No fantasies of your own, the doctors said 5 mg, that means 5 mg.” (6R)* *“I train, I do physiotherapy, they work with me and even if it hurts, nothing happens for a long time, there comes a crucial point and a lot of things get better.” (7R)* *The functionality is different. It’s very related to what you do with your hands and how much you train them.” (29C)*
**Graft integration**	Graft integration characterizes a successful transplantation	*“A successful transplant is when the patient accepts their transplant. (..) That is the first step and the second is when you are ready to deal with the transplant.” (30C)* *“These are my new hands and with these new hands I will continue my new life.” (8R)*

### Pre-transplant factors and preparation

#### Patient selection

One main outcome of this qualitative research was the identification of the role of an interdisciplinary team that carefully optimizes and selects the most suitable patients for UE VCA. For pre-transplant patient selection, it can be noted that a higher level of adherence to protocols and willingness to adapt to an intense training and rehabilitation process are important psychosocial predictors of a successful outcome (2C, 3C). The independent involvement of each discipline in the UE VCA with subsequent interdisciplinary discussion, treatment planning, and task distribution enables a holistic evaluation of the patient, prevents the forgetting of information, and leads to better pre-and post-transplant treatment and overall psychosocial outcomes (4C, 5C).

#### Motives

The decision and motivation to undergo UE VCA transplantation can be influenced by various factors. Motives may include a desire to feel whole again or a desire for (gain of) functionality and sensibility. Presumably, since a certain level of function is achievable with prosthetics, sensibility and the sense of wholeness were more often referenced over functionality (1R, 6C, 2R, 7C, 3R, 8C, 4R, and 9C). The desire to no longer to be perceived as “handicapped” or “disabled” as well as one’s own demands and goals for the future influence the motives for undergoing a UE VCA (6C, 9C).

#### Psychological factors

Psychological factors are not only considered in selection but also contribute to success of UE VCA. Factors that had a positive influence on UE VCA were the pursuit of a regular daily routine, family support, and a high degree of self-discipline (10C, 13C, 14C, 15C). On the other hand, addictive behavior and a lack of cognitive abilities were associated with a worse psychosocial and functional outcome and could be considered as areas to be addressed pre-transplant and/or as relative contraindications (11C, 12C).

### Programmatic factors and influences

#### Clinical resources

Clinical resources and expertise are fundamental to a successful UE VCA transplantation program. An advanced understanding of the field, including literature and exchange with colleagues (16C), and a functional and very experienced transplant team is essential to provide the necessary infrastructure to develop a VCA program (17C).

#### Public perceptions

Public perception of UE VCA transplantation can influence both patients and providers. Patients can be influenced by the public, as they primarily perceive the loss of a hand as something negative (19C). However, a successful UE VCA is often viewed as sensational by the public (19C, 20C). As UE VCA becomes more common and routine, this may reduce the sensationalism and provide more reassurance to the patients (19C, 20C).

### Post-transplant factors and outcomes

#### Rehabilitation

Rehabilitation is particularly important and a lifetime commitment after UE VCA transplantation. Rehabilitation can be positively influenced by a high level of pre-transplant activities of daily living; however, the rehabilitation process can be experienced as a ‘difficult road’ paved with a variety of complications and challenges (21C, 5R, 24C). Physiotherapy and a high degree of self-motivation lead to steady improvements, in terms of sensibility and motor skills, even after several years (22C, 23C, 25C).

#### Follow-up and care

Intense provider involvement in post-transplant follow-up and care increases the likelihood of success. The relationship between the primary healthcare professionals and UE VCA patients differs from ‘regular’ doctor-patient relationships in intensity (26C). Maintaining a close relationship between the primary healthcare professionals and the UE VCA patient is very time consuming and demanding, but due to the often time-sensitive and critical nature of patient issues, it may be necessary to prevent complications (26C, 27C, 28C).

#### Adherence and training

Both adherence and training are big predictors of success. It is essential that patients trust primary healthcare professionals regarding immunosuppressive treatment and other medical management (6R, 7R). Moreover, it is important that patients continue rehabilitation training achieve their highest potential functional improvement (7R, 29C).

#### Graft integration

Graft integration characterizes a successful transplantation. In order to be motivated through intensive rehabilitation, patients do better when they accept grafts as their own hands (30C, 8R).

In summary, the identified important psychosocial factors that lead to a better overall outcome are typically met when a VCA program provides individualized patient-centered and interdisciplinary care.

## Discussion

In this qualitative research on psychosocial predictors and outcomes of patients that underwent UE VCA, three main psychosocial factors have been identified: pre-transplant, programmatic, and post-transplant factors. These psychosocial factors are discussed and contrasted to findings in recent literature.

### Pre-transplant factors and preparation

Numerous other studies have supported the importance of patient selection in optimizing patient outcomes (Brau and Clarke, 2006; Kinsley et al., 2021, 2022). Often overlooked are two key points raised by our patients and teams. Firstly, patient selection is a dynamic not a static process. Longitudinal evaluation and ongoing optimization of a potential candidate’s psychosocial circumstances is fundamental. For example, patients with a history of substance abuse can be appropriately counseled and supported peri-operatively to help them recover without relapse. Secondly, providers have an important role in optimizing patients’ outcomes, ensuring that preparation and support is adequate.

In two separate studies conducted in the US, Kinsley et al. interviewed UE VCA patients, primary caregivers, and healthcare providers to evaluate perceived predictors of transplant access. These included realistic expectations of life after transplantation, strong social support, and positive framing of one’s situation. A deep desire for limbs or an unrealistic expectation of transplant function can both pose a major barrier to accepting a limb transplant that may be imperfect despite intensive rehabilitation and side effects from lifelong immunosuppression. Patients relied heavily on their caregivers and health providers for both physical and emotional support, while expressing the desire to communicate with other transplant recipients to better set expectations.

### Programmatic factors and influences

Programmatic factors have proven challenging for almost all teams globally (Gordon et al., 2009; Gordon and Siemionow, 2009; Kinsley et al., 2021). There are a small number of patients with bilateral upper-extremity amputations who are ready medically and psychosocially for this major intervention. Matching patients who are optimized with teams able to provide the complex care necessary is an ongoing challenge (Gordon et al., 2009; Gordon and Siemionow, 2009; Siemionow and Gordon, 2010; Kumnig et al., 2012, 2013, 2014; Kumnig and Jowsey-Gregoire, 2016; Kinsley et al., 2021).

These large teams also have their own interdisciplinary challenges. We have found that while teamwork is one of the most rewarding aspects of VCA, it can also be one of the most difficult parts. Groups such as the International Society for Vascularized Composite Allotransplantation, the American Society for Reconstructive Transplantation, and the Chauvet Workgroup, all provide collaboration internationally to help educate ourselves within this small field (Jowsey-Gregoire et al., 2016; Kumnig et al., 2022). Furthermore, the International Registry on Hand and Composite Tissue Transplantation provides a superb repository of data that can further encourage collaboration (Kumnig et al., 2022).

In the US, transplant recipients have emphasized how critical it is to have timely access to a dedicated medical care team for long-term wellbeing (Brau and Clarke, 2006). Geographic barriers pose significant logistical challenges for ongoing care, particularly when compounded on financial and compliance issues. These factors are important for healthcare providers during preoperative discussions about continuity of care. Despite the expertise of multidisciplinary programs, providers struggle with setting realistic expectations of rehabilitation and recovery and predicting recipient compliance.

### Post-transplant factors and outcomes

Post-transplantation care is often focused on medication adherence and physical rehabilitation. In our study, we notice the importance of psychosocial support in the follow-up. Rehabilitation is a lifelong commitment after UE VCA, with ongoing steady improvements in sensorimotor function continuing years after transplantation in self-motivated patients who continue their physiotherapy regimens. Maintaining a close relationship between transplant recipients and healthcare providers, although time consuming and demanding, may be necessary to prevent complications, promote adherence to immunosuppression despite adverse effects, and maximize overall functional success.

Similar findings were noted by Kinsley et al. (Brau and Clarke, 2006) in the US. The “intimate” and “special” relationship with the caregiver team was described by some patients as crucial for their practical and emotional needs, particularly during times of feeling alienated. Patients recognized their dependence and the sacrifices they may place on caregivers and care providers, and this recognition may serve as a motivating factor to maximize their independence. The desire for more involved psychological evaluation and therapy was also expressed, congruent with testaments of resilience, positive attitude, purposeful rehabilitation, and strong social supports being favorable psychosocial factors for a good functional outcome.

### Limitations and proposed directions for future research

Future research efforts that are directed at sharing similar evaluation strategies across centers are needed to establish universal guidelines, pathways, and assessments for candidate evaluation and recipient evaluation (Dew et al., 2007; Kumnig et al., 2014). Another important component of interdisciplinary screening should be the identification of at-risk candidates. Intervention strategies to assist these candidates might then lead them to be eligible for this treatment and might especially be beneficial in supporting their ability to succeed with medication adherence and overall QOL post-transplantation (Kumnig et al., 2012, 2014; Kumnig and Jowsey-Gregoire, 2016).

The citations in Table 1 are more originating from VCA healthcare professionals compared to VCA patient families. However, the number of quotations from each group is proportional to the number of study participants from each group: of 12 participants in our study, 4 UE VCA patients, 7 were healthcare professionals, and only 1 was a patient family member. Our clinical observations show that data from patient families are difficult to collect and thus of particular value to the field, but we want Table 1 to be reflective of our actual data pool. We will certainly endeavor to continue collecting qualitative data from patient families in future studies.

## Conclusion

Psychosocial factors are important elements in the assessment and follow-up care for UE VCA. To best capture psychosocial elements of care, protocols must be individualized, patient-centered, and interdisciplinary. Recent research has shown that proposed directions for future research should particularly focus on adherence, training, and close relationship with healthcare providers in the pre-and post-transplant course. The importance of psychosocial factors cannot be overlooked when assessing prospective UE VCA patients preoperatively and optimizing recovery and functional rehabilitation postoperatively. As with all QoL interventions, patients’ subjective experiences are relevant to assessing whether an intervention achieves its aim. Investigating psychosocial predictors and collecting outcomes is, thus, critical to justifying UE VCA as a medical intervention and to providing accurate and salient information to candidates considering the procedure.

## Data availability statement

The raw data supporting the conclusions of this article will be made available by the authors, without undue reservation.

## Ethics statement

The studies involving human participants were reviewed and approved by the Ethics Committee of the Medical University of Innsbruck (vote nr.1044/2020). The patients/participants provided their written informed consent to participate in this study.

## Author contributions

NH: writing the manuscript, performance of the research, and contributed new insights. KZ: writing and editing the manuscript. ST, SK, and MK: participated in research design, wrote the manuscript, performance of the research, and contributed new insights. ZZ: participated in research design and contributed important insights to perform the research and to wrote this manuscript. JK: contributed important insights to perform the research and wrote this manuscript. All authors contributed to the article and approved the submitted version.

## Funding

This work was supported by the US Office of the Assistant Secretary of Defense for Health Affairs under the Reconstructive Transplant Research Program—Qualitative Research Award No. W81XWH-17-1-0400. Opinions, interpretations, conclusions, and recommendations are those of the authors and are not necessarily endorsed by the US Department of Defense.

## Conflict of interest

The authors declare that the research was conducted in the absence of any commercial or financial relationships that could be construed as a potential conflict of interest.

## Publisher’s note

All claims expressed in this article are solely those of the authors and do not necessarily represent those of their affiliated organizations, or those of the publisher, the editors and the reviewers. Any product that may be evaluated in this article, or claim that may be made by its manufacturer, is not guaranteed or endorsed by the publisher.

## Abbreviations

UE VCA: upper-extremity vascularized composite allotransplantation
ISVCA: International Society of Vascularized Composite Allotransplantation
QOL: quality of life
SOT: solid organ transplantation
VCA: vascularized composite allotransplantation
